# The BDNF Val66Met polymorphism regulates glucocorticoid-induced corticohippocampal remodeling and behavioral despair

**DOI:** 10.1038/tp.2017.205

**Published:** 2017-09-19

**Authors:** M Notaras, X Du, J Gogos, M van den Buuse, R A Hill

**Affiliations:** 1 Behavioural Neuroscience Laboratory, Florey Institute of Neuroscience & Mental Health, Parkville, VIC, Australia; 2 Psychoneuroendocrinology Laboratory, Florey Institute of Neuroscience & Mental Health, Parkville, VIC, Australia; 3 Center for Neurogenetics, Feil Family Brain and Mind Research Institute, Weill Cornell Medical College, Cornell University, New York, NY, USA; 4 Department of Psychiatry, Monash University, Melbourne, VIC, Australia; 5 Departments of Biophysics & Neuroscience, Columbia University, New York, NY, USA; 6 School of Psychology and Public Health, La Trobe University, Melbourne, VIC, Australia; 7 Department of Pharmacology, University of Melbourne, Melbourne, VIC, Australia; 8 College of Public Health, Medical and Veterinary Sciences, James Cook University, Townsville, QLD, Australia

## Abstract

The BDNF Val66Met polymorphism has been associated with sensitivity to stress and affective disorders. We therefore sought to model the inter-causality of these relationships under controlled laboratory conditions. We subjected humanized BDNF Val66Met (hBDNF^Val66Met^) transgenic mice to a history of stress, modeled by chronic late-adolescent corticosterone (CORT) exposure, before evaluating affective-related behavior using the forced-swim test (FST) in adulthood. While hBDNF^Met/Met^ mice had a depression-like phenotype in the FST irrespective of CORT, hBDNF^Val/Val^ wildtype mice had a resilient phenotype but developed an equally robust depressive-like phenotype following CORT. A range of stress-sensitive molecules were studied across the corticohippocampal axis, and where genotype differences occurred following CORT they tended to inversely coincide with the behavior of the hBDNF^Val/Val^ group. Notably, tyrosine hydroxylase was markedly down-regulated in the mPFC of hBDNF^Val/Val^ mice as a result of CORT treatment, which mimicked expression levels of hBDNF^Met/Met^ mice and the FST behavior of both groups. The expression of calretinin, PSD-95, and truncated TrkB were also concomitantly reduced in the mPFC of hBDNF^Val/Val^ mice by CORT. This work establishes BDNF^Val66Met^ genotype as a regulator of behavioral despair, and identifies new biological targets of BDNF genetic variation relevant to stress-inducible disorders such as depression.

## Introduction

Affective disorders, such as major depressive disorder, have an estimated lifetime prevalence of ~20.8%,^1^ and are projected to become a leading cause of long-term disability.^2^ The neurobiology of affective disorders has advanced rapidly, and has led to the identification of a number of core pathology that includes reduced neurotrophic support and synaptic remodeling^3^ within brain regions such as the hippocampus^4, 5^ and medial prefrontal cortex (mPFC).^5^

One environmental factor that has consistently been associated with the onset and maintenance of affective disorders is stress. Clinical markers of stress exposure, such as daily life stress, history of stressful life events, and trauma have all been shown to play a role in aspects of affective disorder symptomology or risk, and collectively support the assertion that both developmental and ongoing stress are capable of inducing depressive disorders.^6^ In rodent models, exposure to a broad range of stress paradigms has led to the identification of several remodeling events that putatively occur as a result of glucocorticoid stress hormones within the brain. These include widespread alterations within the hippocampus, including reductions in dendritic spine branching and complexity,^7^ changes in the expression of brain-derived neurotrophic factor (BDNF),^8^ NMDA receptor subunit reorganization^9^ and synaptic scaffolding proteins such as the excitatory postsynaptic molecule PSD-95^10^ and presynaptic marker synaptophysin.^11^ A similar pattern of reorganization following stress also occurs in other brain regions including the mPFC, which appears particularly vulnerable to stress-induced alterations in noradrenergic activity^12, 13^ and the maturation of inhibitory interneuron networks.^14^

BDNF has been widely studied as a susceptibility factor for both stress and affective dysregulation. BDNF plays a fundamental role in brain development, neuronal differentiation and synaptic plasticity.^15^ It has been suggested that BDNF is a transducer of antidepressant effects,^16^ principally because BDNF is recruited by antidepressant therapeutics (as well as other mood disorder treatments such as electroconvulsive shock^17^ and transcranial magnetic stimulation therapies^18^) and is suppressed by many risk factors for mood disorders including stress.^19^ In rodent models, BDNF also mediates behavioral endophenotypes of relevance to affective disorders,^20, 21^ while in clinical samples serum BDNF concentrations predict the effectiveness of selective-serotonin reuptake inhibitors in the treatment of depression.^22^

The BDNF Val66Met polymorphism, named after a Valine→Methionine substitution at codon 66 within the BDNF prodomain, has been widely studied as a risk factor for affective disorders due to its common frequency and established functionality.^15^ Specifically, the Val66Met substitution results in the diminished activity-dependent release of BDNF,^23^ deficient hippocampus-dependent memory function^24^ and a lack of response to antidepressant therapeutics in BDNF^Val66Met^ knock-in mice.^24, 25^ However, the role of this gene variant as a risk factor for mood disorders and modulator of antidepressant response has been the source of much controversy given non-concordant results between association studies (see ref. 15 for extensive review). These inconsistent clinical findings strengthen the case for animal models in providing well controlled findings on the biological mechanisms which underpin stress responsivity, as well as antidepressant response. That said, a number of reports have emerged in recent years which suggest that the Val66Met variant may induce HPA axis dysfunction,^25^ which we previously hypothesized may lead to a long-lasting sensitivity to glucocorticoid stress hormones^15^ and thus vulnerability to affective dysregulation. In support of this hypothesis, it has been previously published that childhood adversity may unmask an effect of the 66Met allele on depression,^26^ while in otherwise healthy adults a history of sexual trauma has been shown to moderate the expression of depressive symptoms among 66Met allele carriers.^27^ Of concern, however, is a report which suggests that first-episode depression patients that carry BDNF^Val/Met^ and BDNF^Met/Met^ genotypes are more likely to have experienced stressful life events than BDNF^Val/Val^ genotype controls,^28^ suggesting that this variant may induce a stress-sensitivity loop whereby 66Met allele carriers are not just more sensitive to the effects of stress but are also more likely to experience stressful life events.

Therefore, we sought to resolve what effect BDNF^Val66Met^ genotype has on corticohippocampal molecular remodeling and depression-related behavioral despair using the forced-swim test (FST), and whether a phenotype was dependent on, or unmasked by, a history of chronic stress.

## Materials and methods

### Genetic construct of humanized BDNF^Val66Met^ mice

The BDNF gene is highly conserved between species,^29^ and despite differences in promoter structure the coding exon of mouse *Bdnf* closely resembles that of human *BDNF*. To generate the Val66Met knock-in human-BDNF expressing (hitherto referred to as hBDNF^Val66Met^) mice, a 274bp region was amplified from one BDNF^Val/Val^ and one BDNF^Met/Met^ human donor and inserted into the mouse BDNF gene, replacing the respective murine sequence.^30^ Extensive procedural details (including details of recombination probes and targeting constructs) are available in a previous publication.^30^ Once generated, BDNF^Human Val/+^ mice were crossed with BDNF^Human Met/+^ mice to generate hBDNF^Val/Met^ mice, which were subsequently used as breeders to produce hBDNF^Val/Val^ and hBDNF^Met/Met^ mice. All mice were maintained on a C57BL/6 background. Mice were bred and group-housed in individually ventilated cages under standard lighting conditions (12 h light cycle) and provided *ad libitum* access to food and water. All experimental procedures were approved by the Florey Institute of Neuroscience’s animal ethics committee, and conducted in accordance with guidelines set by the National Health and Medical Research Council of Australia.

### Late-adolescent chronic CORT protocol

Chronic adolescent stress was simulated by treating pseudorandomly selected mice with 25 mg l^−1^ of the mouse stress hormone, corticosterone (CORT), which was dissolved in the animal’s drinking water, as previously described.^31^ This dosage was selected based on previous work which has shown that this low-dosage recapitulates depression-like brain and behavioral phenotypes without peripheral side-effects that may influence animal health.^32^ Importantly, chronic CORT delivered via drinking water also recapitulates several translationally relevant aspects of daily life stress, as it follows a diurnal cycle and is capable of inducing persistent alterations in anxiety- and affective-related behavior that can be rescued by antidepressant treatment.^32^ This model thus allows the long-term evaluation of stress-induced changes in brain chemistry or behavioral outputs that last beyond the disruption of the HPA axis during treatment,^32, 33^ as is similar to the lasting effects of early life stress on the chronicity of depression-related symptoms in humans.^34^ Animals assigned to the chronic CORT group received the treatment solution between weeks six to nine, a developmental period which is similar to late adolescence in humans as evidenced by a late spike in the production of sex-steroid hormones.^35^ Mice allocated to the control group received unaltered water over this timeframe. There was a two-week washout period following treatment, so that the HPA axis and circulating glucocorticoids levels could recover,^33^ before behavioral testing commenced.

### Forced-swim test

The forced-swim test was used as our representative model of learned helplessness and behavioral despair. Mice were ~15 weeks of age at testing. Four swim chambers (maximum volume of 2 l) were filled with 1.7 l of water pre-heated to 21 °C, and were separated by white dividers. Before experimentation, mice were habituated for one hour in the test room. Following habituation, mice were individually placed into a swim chamber and allowed to swim for six minutes. Behavior was recorded for offline analysis, with time spent immobile – defined as a lack of swimming movement of a duration equal to or exceeding one second – being quantified by two observers who were unaware of group allocations. The primary outputs were latency to immobility, the time-course of immobility over the test session and the average immobility over the last 4 minutes of testing.

### Preparation of brain lysates and western blot

Experimental mice were killed via cervical dislocation one week after completing behavior. Brains were snap-frozen on dry ice, stored at −80 °C and later dissected with a brain matrix according to the Paxinos and Watson atlas.^36^ The dorsal hippocampus (DHP), ventral hippocampus (VHP) and mPFC (which included the prelimbic, infralimbic and cingulate cortices) were chosen as our primary regions of interest given their involvement in mood disorders. Once dissected, protein was extracted and aliquoted into 50 μg protein samples before undergoing SDS–PAGE and transfer to nitrocellulose membrane, as previously described.^31^ Membranes were incubated in 5% bovine serum albumin in Tris-buffered saline with primary antibody overnight (β-Actin: 1:10000, Sigma-Aldrich, Sydney, NSW, Australia; Calretinin: 1:1000, Swant, Switzerland cr7697; fl. and tr.TrkB: 1:1000, Santa Cruz Biotechnology, Dallas, TX, USA H181; pTrkB^Y515^: 1:1000, Abcam, Cambridge, UK ab109684; Gephyrin: 1:1000, Abcam ab32206; NR2A: 1:1000, Abcam ab14596; NR2B/NR1: 1:200, Abcam ab110; PSD-95: 1:1,000, Abcam ab18258; Synaptophysin: 1:400, Sigma-Aldrich s5768; Tyrosine Hydroxylase: 1:1,000, Millipore, Bayswater, VIC, Australia ab152). Blots were imaged using a LAS-4000 Luminescence Analyzer (Fuji Film Life Science, Stamford, CT, USA) and analyzed using the TotalLab Quant Analysis Software (Total Lab, Newcastle, UK). Western blots were repeated between two to four times to confirm results.

### BDNF Enzyme-Linked immunosorbent assay

BDNF expression was quantified using the BDNF Emax ImmunoAssay System (Promega, Madison, WI, USA), so that an exact concentration per region could be derived between hBDNF^Val66Met^ genotype groups. According to the manufacturer, the kit has a sensitivity as low as 15.6 pg μl^−1^, with <3% cross-reactivity to other neurotrophins. Assays were performed according to manufacturer’s instructions. Briefly, a 96-well plate was coated with 100 μl of anti-BDNF antibody diluted in carbonate-coating buffer (10 μl:9.99 ml) and incubated overnight. The following morning, plates were blocked for 1 h with supplied buffer before 100 μl of BDNF standards and experimental samples were plated in duplicate. After incubating with shaking for 2 h, 100 μl of an anti-BDNF polyclonal antibody solution (1:500 diluted in blocking buffer) was added per well and incubated for a further 2 h. Following washing, 100 μl of diluted anti-IgY HRP conjugate (1:200 in blocking buffer) was added to wells and incubated for 1 h. Absorption was correspondingly developed by adding 100 μl of supplied TMB solution per well and and incubating for 10 min, and terminated with 100 μl per well of 1 N hydrochloric acid. Absorption was read at 450 nm using a plate reader.

### Statistical analysis

The total sample size of the current study was 166 hBDNF^Val66Met^ mice, comprising 26 control and 25 CORT-treated hBDNF^Val/Val^ mice, 40 control and 28 CORT-treated hBDNF^Val/Met^ mice, and 23 control and 24 CORT-treated hBDNF^Met/Met^ mice. Sampling was based on our prior investigations using this mouse line, which have been adequately powered to detect medium-to-large as well as more subtle effects upon pooling (see below; refs. 31, 37). Data analysis was undertaken using the IBM SPSS and Graphpad Prism packages. For tests that only involve between-group comparisons a 3 (genotype) × 2 (sex) × 2 (treatment) Analysis of Variance (ANOVA) was conducted, with assumptions being screened for. Within-group comparisons were analyzed using a Mixed Model ANOVA. SEMs were used as our measure of variance for all graphing. As no significant interaction involving the main effect of sex was observed on any given behavioral or molecular measure, denoting no effect of sex on the main effects of genotype and treatment, data from male and female mice were analyzed together to increase power as consistent with previous investigations.^31, 37^ No exclusion criteria were applied, except for outliers (defined as values falling outside of ± 2 s.d.). Statistical significance was set at *P*=0.05, as per Fisher’s tables, while all between-group comparisons were corrected for multiple comparisons using Tukey’s or Holm-Sidak’s method depending on observed power.

## Results

### hBDNF^Val66Met^ genotype determines vulnerability to stress-related despair

The FST was used as our model of behavioral despair. Analysis revealed main effects of hBDNF^Val66Met^ Genotype (*F*(2,160)=9.7, *P*=0.0001) and chronic CORT (*F*(1,160)=17.5, *P*<0.0001), as well as their interaction (*F*(2,160)=5.0, *P*=0.0077), on immobility. Post-hoc comparisons of the significant main effects revealed that mice carrying the hBDNF^Met/Met^ genotype were immobile for significantly longer than hBDNF^Val/Val^ mice at baseline (*P*<0.0001), while mice treated with CORT were also immobile for significantly longer than those allocated to the control group (*P*<0.0001). Post-hoc analysis of the genotype × treatment interaction revealed that the chronic CORT treatment selectively increased the immobility of hBDNF^Val/Val^ mice relative to controls (*P*<0.0001). No effect of CORT was detected among the other genotype groups. No significant differences were observed between groups for latency to immobility.

### Late-adolescent CORT exposure suppresses tyrosine hydroxylase and calretinin expression in the mPFC of Adult hBDNF^Val/Val^ Mice

Expression of tyrosine hydroxylase (TH), the rate-limiting enzyme involved in the biosynthesis of catecholamine neurotransmitters such as noradrenaline,^38^ has been implicated in suicidality,^39^ is altered in depression^40, 41^ and is responsive to antidepressant therapeutics.^42^ In addition, gene variants within the TH gene have also been associated with mood disorder symptomology,^43^ while chronic stress may regulate the release of noradrenaline within the hippocampus^44^ and prefrontal cortex.^12, 45^ To this end, we examined whether expression of TH was altered in our three regions of interest in our hBDNF^Val66Met^ mouse line and whether a history of chronic CORT treatment alters the expression of this enzyme in adulthood. There was no effect of hBDNF^Val66Met^ genotype or history of adolescent CORT exposure on the expression of TH in the DHP or VHP. However, within the mPFC a significant hBDNF^Val66Met^ genotype × history of CORT treatment (*F*(1, 49)=4.42, *P*=0.041) interaction was observed, whereby chronic CORT reduced the expression of TH in hBDNF^Val/Val^ mice by >50% (*P*<0.001) to levels that were consistent with vehicle and CORT-treated hBDNF^Met/Met^ mice, which did not differ from one another. A further main effect of CORT was also detected in the mPFC (*F*(1, 49)=10.6, *P*=0.002), however no other main effects reached significance.

While TH expression in the mPFC largely represents terminal TH present in projections from the midbrain, there also remains a subset of cortical TH-immunoreactive cells that are predominantly bipolar type inhibitory interneurons that selectively express the calcium-channel binding protein calretinin.^46^ As such, we next examined whether the expression of calretinin was altered across our regions of interest. There were no significant group differences in the DHP or VHP. However, in the mPFC a hBDNF^Val66Met^ genotype × CORT treatment (*F*(1, 49)=4.54, *P*=0.038) interaction was once more observed where, like TH, the expression of calretinin was selectively decreased among the hBDNF^Val/Val^ group as a function of CORT treatment (*P*<0.05). No further main effects or interactions reached significance.

### No Effect of hBDNF^Val66Met^ Genotype on Basal BDNF Levels in Corticohippocampal Regions

BDNF levels were quantified via ELISA so to determine an absolute concentration of basal BDNF between groups. BDNF expression in the DHP was not significantly different between hBDNF^Val/Val^ and hBDNF^Met/Met^ mice, however a significant effect of prior CORT treatment was detected in this brain region (*F*(1,30)=13.73, *P*=0.0009) reflecting that this treatment increased BDNF expression irrespective of genotype. BDNF expression in the VHP and mPFC did not significantly differ between the various treatment and genotype groups. No other main effects or comparisons reached significance.

### Region-Specific Effects of hBDNF^Val66Met^ Genotype and CORT on TrkB Receptors

We also screened for an effect of hBDNF^Val66Met^ genotype and history of CORT treatment on the expression of BDNF’s cognate receptor TrkB. This included assessments of the functional full-length TrkB (fl.TrkB) receptor, the catalytic domain-lacking and dominant-negative truncated TrkB (tr.TrkB) receptor,^47, 48^ and the phosphorylation of TrkB^Y515^. In the DHP, a significant effect of hBDNF^Val66Met^ genotype was detected for fl.TrkB (*F*(1, 52)=8.13, *P*=0.0062). Post-hoc testing revealed that hBDNF^Met/Met^ mice had higher fl.TrkB expression than hBDNF^Val/Val^ mice in this brain region (*P*<0.01). While this genotype effect appeared to be moderated by an inhibitory effect of CORT on the expression of TrkB protein in the DHP of hBDNF^Val/Val^ mice, there was no statistical support for a genotype × CORT interaction (*P*=0.10) in spite of our relatively large group sizes (in this region, per group, *n*=14). Contrary to this, fl.TrkB was not altered in the VHP or mPFC. The analysis of tr.TrkB receptor expression revealed a significant main effect of chronic CORT treatment (*F*(1, 51)=9.48, *P*=0.0033) in the DHP, which decreased tr.TrkB expression irrespective of genotype in this region. In the VHP, a significant main effect of hBDNF^Val66Met^ genotype (*F*(1, 52)=4.7, *P*=0.035) emerged, whereby hBDNF^Met/Met^ mice had lower expression of the tr.TrkB isoform than hBDNF^Val/Val^ mice (*P*<0.05). In the mPFC, a significant main effect of chronic CORT treatment (*F*(1, 48)=6.03, *P*=0.018), as well as a hBDNF^Val66Met^ genotype × chronic CORT treatment interaction (*F*(1, 48)=5.57, *P*=0.022) emerged for tr.TrkB availability. Post-hoc analysis revealed that while chronic CORT treatment tended to decrease the expression of tr.TrkB irrespective of genotype, the magnitude change in tr.TrkB expression following CORT treatment was more pronounced among hBDNF^Val/Val^ mice (*P*<0.01). Lastly, the only main effect to reach significance for pTrkB^Y515^ was a history of CORT treatment, which emerged in the DHP (*F*(1, 51)=5.74, *P*=0.0203), VHP (*F*(1, 52)=22.51, *P*<0.0001) and mPFC (*F*(1, 40)=8.67, *P*=0.0054). Chronic CORT increased basal pTrkB^Y515^, which was normalized to fl.TrkB levels, in these regions.

### hBDNF^Val/Val^ Mice are more vulnerable than hBDNF^Met/Met^ mice to synaptic protein reorganization following chronic CORT exposure

To assess the effect of hBDNF^Val66Met^ genotype and chronic CORT exposure on synaptic integrity, expression levels of the excitatory and inhibitory terminal scaffolding proteins PSD-95 and gephyrin, as well as the presynaptic vesicle transport molecule, synaptophysin, were screened in the DHP, VHP and mPFC. In the DHP, a significant hBDNF^Val66Met^ genotype x CORT interaction was observed for the expression of synaptophysin (*F*(1, 43)=4.34, *P*=0.043), whereby chronic CORT reduced its expression in the DHP of hBDNF^Val/Val^ mice but not hBDNF^Met/Met^ mice (*P*<0.01). In addition to this, the expression of PSD-95 was found to significantly depend on hBDNF^Val66Met^ genotype in the DHP (*F*(1, 50)=18.66, *P*<0.0001); reflecting that hBDNF^Met/Met^ mice had higher expression levels of PSD-95 than hBDNF^Val/Val^ mice in this region. A history of chronic CORT exposure in adolescence also significantly decreased the expression of PSD-95 (*F*(1, 50)=13.76, *P*=0.0005), but did not interact with genotype. Similarly, no effect of hBDNF^Val66Met^ genotype was detected on the expression of gephyrin in the DHP, which was reduced irrespective of genotype by history of CORT exposure (*F*(1, 52)=9.21, *P*=0.0037). In the VHP, none of the main effects reached significance nor did they interact to determine the expression of synaptophysin, PSD-95 or gephyrin, suggesting that the DHP but not VHP is selectively sensitive to the effect of hBDNF^Val66Met^ genotype and the long-term effect of chronic adolescent CORT treatment. In the mPFC, none of the main effects reached significance for the expression of our synaptic proteins of interest. However, a subtle but significant hBDNF^Val66Met^ genotype × chronic CORT treatment interaction emerged for the expression of PSD-95 (*F*(1, 48)=6.12, *P*=0.017). Post-hoc testing revealed that, once more, it was the hBDNF^Val/Val^ wildtype genotype which was associated with reduced PSD-95 expression following the chronic CORT treatment (*P*<0.05). All other comparisons failed to reach significance.

### hBDNF^Val66Met^ genotype does not alter NMDA receptor subunit expression

Given the role of PSD-95 as a scaffolding protein involved in securing NMDA receptors to the cell membrane,^49^ that BDNF^Val66Met^ genotype is associated with altered NMDA receptor physiology^50, 51, 52^ and that NMDA receptor subunits may modulate FST performance,^53, 54, 55^ we next screened for differences in expression of the NMDA receptor NR2A, NR2B and NR1 subunits. In the DHP, there was a main effect of chronic CORT treatment on NR2A (*F*(1, 52)=4.73, *P*=0.034), NR2B (*F*(1, 52)=4.06, *P*=0.049) and NR1 (*F*(1, 46)=7.97, *P*=0.007) subunit expression, where a history of CORT decreased the expression of these subunits irrespective of genotype. In the VHP a main effect of CORT was observed for NR2A (*F*(1, 52)=11.55, *P*=0.001) and NR2B (*F*(1, 51)=19.27, *P*<0.0001) expression, albeit in the opposite direction to the effect seen in the DHP. No effect of hBDNF^Val66Met^ genotype or history of CORT treatment emerged for NR1 within the VHP. Lastly, in the mPFC, the only effect to reach significance was that of CORT on the expression of the NR2B subunit (*F*(1, 52)=17.25, *P*=0.0001), where expression was increased as a result of CORT treatment irrespective of hBDNF^Val66Met^ genotype. No other main effects or interactions reached significance.

## Discussion

**Figure 1:**
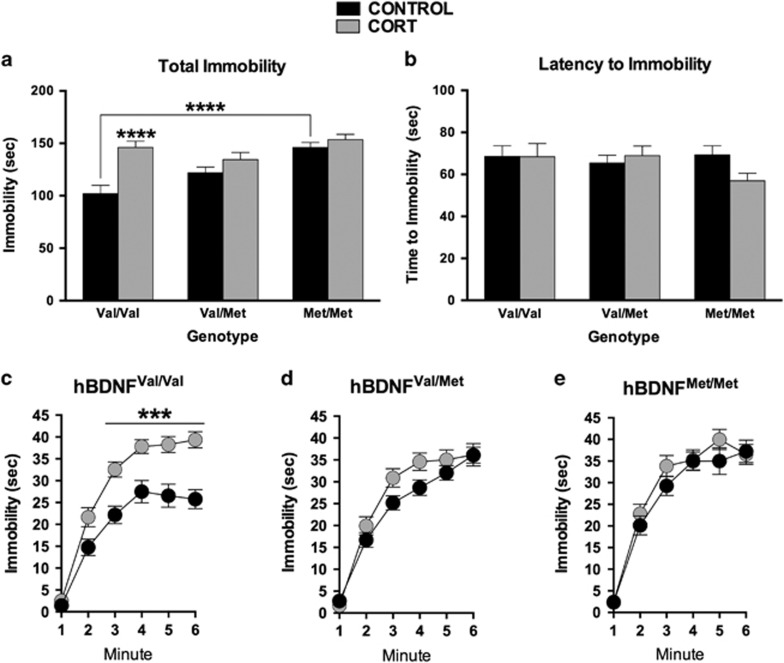
hBDNF^Val66Met^ genotype and history of CORT treatment regulate immobility in the Forced-Swim Test (FST). (**a**) A main effect of genotype was detected at baseline for immobility, where hBDNF^Met/Met^ mice were immobile for significantly longer than hBDNF^Val/Val^ mice. An interaction between genotype and CORT treatment was also observed, where the hBDNF^Val/Val^ mice were most susceptible to the effects of CORT on immobility. (**b**) No main effect or interaction was detected for average latency to immobility. (**c**–**e**) An analysis of immobility as a time-course confirmed the selective effect of CORT on this genotype group. All data are presented as mean±s.e.m.; ***P<0.001, ****P<0.0001. Per group, *n*=23–40.

The aim of the current study was to examine the effect of the BDNF Val66Met polymorphism on behavioral despair at baseline, and whether a depression-like phenotype was unmasked or modulated by a history of chronic stress. The FST was used as our representative paradigm for behavioral despair, while a CORT exposure paradigm was chosen as our model of stress due to its specificity in receptor action in the brain and diurnal cycle which recapitulates the core features of chronic life stress. Our data supported our hypothesis that the hBDNF^Val66Met^ genotype gates learned helplessness in adulthood, and that a history of CORT exposure during late adolescence acts to regulate this effect. Specifically, we report that while hBDNF^Met/Met^ mice have a robust learned helplessness phenotype at baseline, following chronic CORT the hBDNF^Val/Val^ genotype group develops a behavioral despair phenotype that is just as robust as that of the hBDNF^Met/Met^ group (Figure 1).

The presence of a 66Met-derived despair phenotype at baseline in our hBDNF^Val66Met^ model is consistent with the broader literature on the role of BDNF in animal models of depression, where deficient BDNF expression results in social defeat and learned helplessness phenotypes,^20, 21^ and likely occurs via the disrupted activity-dependent release of BDNF induced by the 66Met substitution. In favor of the construct validity of this model, our behavioral data are also consistent with clinical data from population genetic studies which have reported evidence that the 66Met allele may be associated with affective disorders, as well as clinical components of these disorders.^15^ However, this clinical evidence has been inconsistent,^15^ possibly due to the involvement of interaction factors such as vulnerability to stress. In this respect, our observation that this enduring learned helplessness phenotype occurs at baseline our hBDNF^Met/Met^ genotype group, but that the hBDNF^Val/Val^ wildtype genotype produces a convergent phenotype following a history of simulated stress exposure, provides support for a role of the BDNF^Val66Met^ variant in mood disorders, while also providing an explanation for why results may have failed to replicate between some studies (as a long-term effect of prior stress or trauma may not have been stratified as covariates). In addition to this, the current data also extend our prior report using this mouse line and model of stress^31^ by establishing that glucocorticoid stress hormones differentially interact with BDNF^Val66Met^ genotype to alter behavior depending on the system being studied and construct being measured. Specifically, we previously replicated a gene-dosage effect of the hBDNF^Met/Met^ genotype on fear conditioned memory and short-term spatial memory which we found could be ‘rescued’ by chronic CORT,^31^ while in other behavioral paradigms, such as prepulse inhibition, chronic CORT exposure unveiled a heterozygote disadvantage phenotype among hBDNF^Val/Met^ mice relative to both homozygous genotype groups.^37^ In this respect, we can confirm that for behavioral despair on the FST an effect of hBDNF^Val66Met^ genotype emerges at baseline, and that chronic CORT exposure induces a deficit among hBDNF^Val/Val^ mice, while having no further effect on hBDNF^Val/Met^ mice or hBDNF^Met/Met^ mice (see Figure 1). Thus, hBDNF^Met/Met^ mice perform on the FST similarly to CORT-treated hBDNF^Val/Val^ mice, implicating that the Val66Met variant induces vulnerability to behavioral despair.

**Figure 2:**
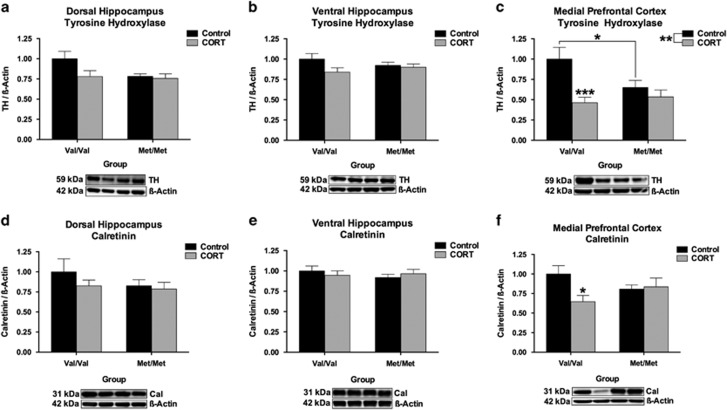
hBDNF^Val66Met^ genotype and chronic CORT exposure interact to selectively reduce the expression of Tyrosine Hydroxylase (TH) and calretinin in the mPFC but not hippocampus. While there was no effect of genotype or chronic CORT treatment on TH (**a, b**) or calretinin (**d, e**) expression in the DHP or VHP, the expression of both TH (**c**) and calretinin (**f**) was found to be reduced in hBDNF^Val/Val^ mice following CORT in the mPFC. All data are presented as mean±s.e.m.; *P<0.05, **P<0.01, ***P<0.001 and ****P<0.0001. Per group, *n*=13–14.

Our investigation of the mechanism that underscores this convergence in FST phenotype between hBDNF^Met/Met^ and CORT-treated hBDNF^Val/Val^ mice revealed several differences in the long-term molecular adaptation to chronic CORT treatment between hBDNF^Val/Val^ and hBDNF^Met/Met^ mice. Specifically, in the mPFC, a history of chronic CORT treatment decreased the expression of TH in hBDNF^Val/Val^ mice to levels consistent with the hBDNF^Met/Met^ genotype group (see Figure 2c), which inversely corresponded with the FST performance of both genotype groups. TH within the cortex is believed to largely originate from terminals of catecholamine projections that emanate from within the midbrain. These projections originate from brain regions responsible for producing dopamine and noradrenaline, which have both been implicated in the adaptation to stress^56^ and mood disorders.^57^ Therefore, our TH phenotype may be related to both dopamine and/or noradrenaline activity. However, as BDNF^+/Met^ mice have increased noradrenergic transporter expression within the locus coeruleus,^25^ and considering that the noradrenergic agent desipramine (but not the SSRI fluoxetine) rescues the FST phenotype of BDNF^+/Met^ mice following restraint stress,^25^ the possibility that our TH phenotype is linked to alterations in noradrenergic activity remains a tantalizing possibility. Noradrenaline plays an important role in modulating the behavioral response to stress, and it is of note that various stress treatments increase noradrenaline release^12^ and sensitization^58, 59^ within the mPFC. Importantly, TH is found within noradrenergic terminals in the PFC,^60, 61^ and selective noradrenaline reuptake inhibitors (but not selective serotonin reuptake inhibitors) reduce Fos-like immunoreactivity in the mPFC following testing on the FST.^62^ As hyporeactivity of the mPFC in response to stress may provide a foundation for stress-related behavioral traits,^63^ it would be of interest to consider measurements of catecholamine activity within the mPFC of hBDNF^Val66Met^ mice, both at baseline and following stress, in future studies. That said, other than neuronal terminal TH, a subset of TH-immunoreactive cells may also exist in the PFC^64^ and these cells are believed to be inhibitory interneurons based on their morphology and colocalization with both GAD and calretinin.^46^ The functionality of these neurons is unknown.^46^ Nevertheless, we also quantified calretinin within the mPFC and found it too was concomitantly reduced selectively in the hBDNF^Val/Val^ genotype group as a result of CORT treatment (Figure 2f). As such, it is possible that the significant reduction in TH within the mPFC of CORT-treated hBDNF^Val/Val^ mice may arise from an alteration in terminal TH and noradrenergic innervation by ascending projections, a selective loss of TH-immunoreactive calretinin interneurons within the mPFC, or a combination of both outcomes.

**Figure 3:**
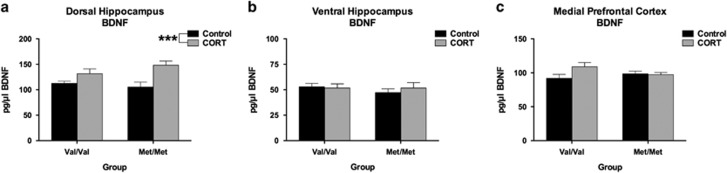
No effect of hBDNF^Val66Met^ genotype on basal BDNF protein levels in the (**a**) DHP, (**b**) VHP or (**c**) mPFC. The only main effect to reach significance in these regions was a history of chronic CORT treatment in the DHP, which increased BDNF expression levels independent of hBDNF^Val66Met^ genotype. All data are presented as mean±s.e.m.; ***P<0.001. Per group, *n*=8–11.

**Figure 4:**
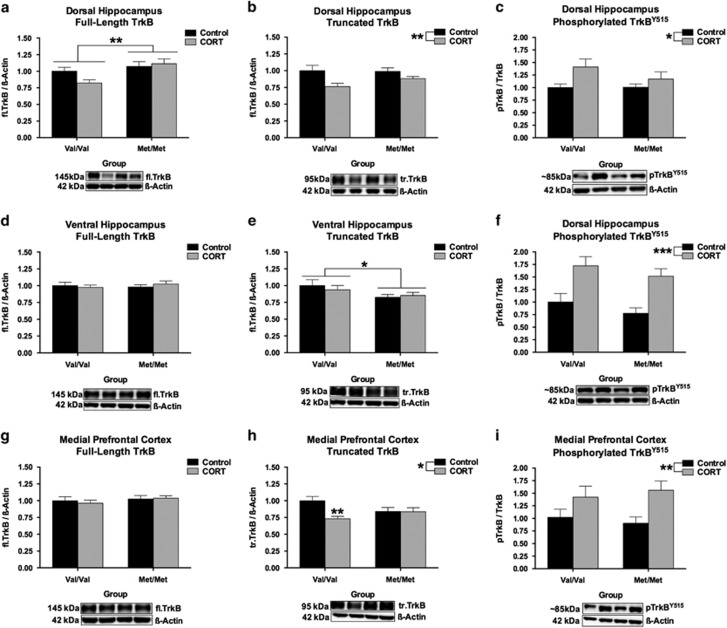
Effects of hBDNF^Val66Met^ genotype and prior CORT exposure on TrkB isoforms. (**a**) hBDNF^Met/Met^ mice have (subtly) higher fl.TrkB expression relative to hBDNF^Val/Val^ mice in the DHP, while chronic CORT tends to (**b**) decrease tr.TrkB expression while (**c**) increasing TrkB^Y515^ phosphorylation in this brain region. In the VHP, (**d**) fl.TrkB was unchanged however (**e**) hBDNF^Met/Met^ mice tended to have lower tr.TrkB expression than hBDNF^Val/Val^ mice. (**f**) CORT treatment increased pTrkB^Y515^ phosphorylation in the VHP. In the mPFC, (**g**) fl.TrkB was once more unchanged. (**h**) However, a significant genotype × treatment interaction emerged for tr.TrkB expression whereby hBDNF^Val/Val^ mice selectively responded to chronic CORT. Following CORT, expression levels of tr.TrkB in the mPFC of hBDNF^Val/Val^ mice were reminiscent of those observed in the mPFC of hBDNF^Met/Met^ mice. (**i**) Lastly, as in the DHP and VHP, a history of CORT also increased pTrkB^Y515^ phosphorylation in the mPFC. All data presented as mean±SEM; *P<0.05, **P<0.01, and ***P<0.001. Per group, *n*=9–14.

**Figure 5:**
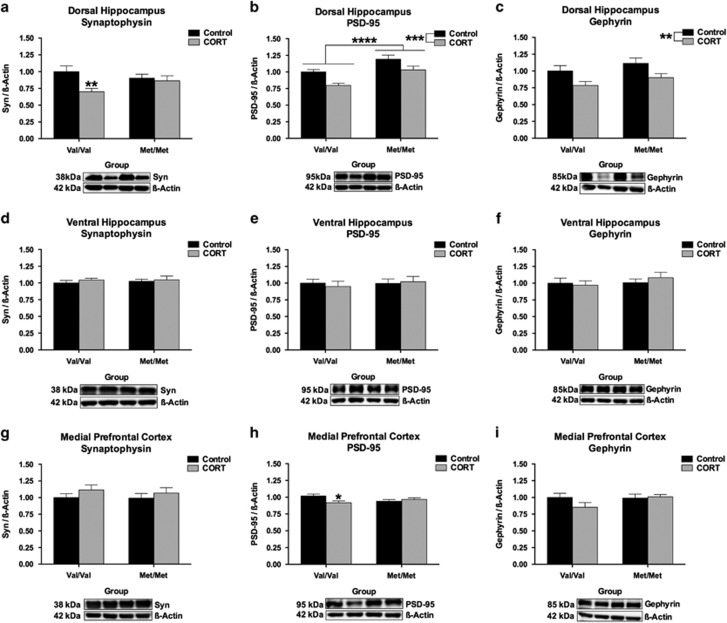
hBDNF^Val66Met^ genotype alters synaptic protein expression within the DHP and mPFC but not VHP. (**a**) Within the DHP, the expression of synaptophysin was labile to remodeling by chronic CORT as a function of hBDNF^Val66Met^ genotype, where hBDNF^Val/Val^ mice were selectively vulnerable to the long-term effect of CORT. (**b**) The expression of PSD-95 in the DHP was found to be dependent on hBDNF^Val66Met^ genotype. hBDNF^Met/Met^ mice had significantly higher PSD-95 expression, an excitatory post-synaptic marker and scaffolding protein, than hBDNF^Val/Val^ mice. While an effect of CORT on the expression of PSD-95 in the DHP was also detected, which decreased the expression of this scaffolding protein, this effect was independent of genotype. (**c**) This effect of CORT was recapitulated for the expression of inhibitory terminal marker gephyrin. There were no significant differences in synaptic protein reorganization in the VHP (**d-f**) In the mPFC, there were no detectable alterations in the expression of synaptophysin (**g**) or gephyrin (**i**), however a subtle hBDNF^Val66Met^ × chronic CORT treatment interaction emerged for the expression of PSD-95 which resulted in a selective decrease of this marker among the hBDNF^Val/Val^ group as a consequence of CORT treatment (**h**). All data are presented as mean±SEM; *P<0.05, **P<0.01, ***P<0.001, and ****P<0.0001. Per group, *n*=13–14.

**Figure 6:**
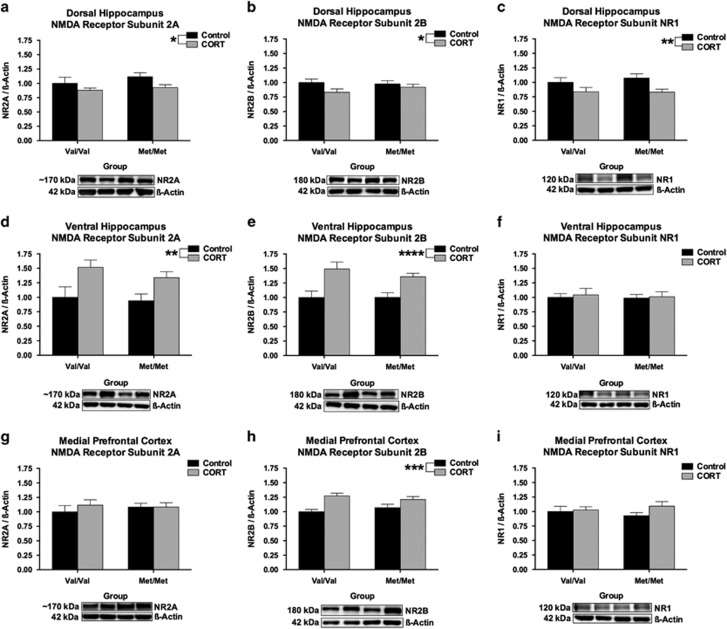
Chronic CORT alters the expression of the NMDA NR2A, NR2B and NR1 subunit composition independent of hBDNF^Val66Met^ genotype. Chronic CORT treatment was found to decrease (**a**) NR2A, (**b**) NR2B and (**c**) NR1 subunit expression in the DHP. In the VHP, chronic CORT increased the expression of (**d**) NR2A and (**e**) NR2B but had no effect on the expression of (**f**) NR1. In the mPFC, the only group difference to emerge was an effect of CORT on (**h**) NR2B, which also increased the expression of this subunit. No modulatory effect of hBDNF^Val66Met^ genotype emerged for the (**g**) NR2A or (**i**) NR1 subunits in this region. All data presented as mean±s.e.m.; *P<0.05, **P<0.01, ***P<0.001, and ****P<0.0001. Per group, *n*=13–14.

Importantly, long-lasting changes in basal BDNF expression following CORT does not appear to underscore these behavioral and molecular phenotypes. The DHP was the only structure that exhibited a change in BDNF expression across our groups (Figure 3a), where an indiscriminate effect of prior CORT exposure increased total BDNF. While it is unclear why only the DHP exhibited this shift in expression, it is possible that the DHP is developmentally more sensitive to the effects of CORT than the VHP. This explanation is consistent with genotype-specific alterations in the developmental expression of GRs within the DHP of hBDNF^Val66Met^ mice, as we have previously reported (see supplementary data of ref. 31), which establishes that differences in the developmental trajectory of the hippocampal longitudinal axis may result in critical periods of vulnerability to stress. Likewise, neither fl.TrkB expression nor phosphorylation (Figure 4) appeared to be associated with the behavioral and molecular phenotypes reported here. However, tr.TrkB receptors were found to be selectively down-regulated in the mPFC of hBDNF^Val/Val^ mice following CORT (Figure 4h). As tr.TrkB receptors have dominant-negative effects upon fl.TrkB signaling,^47, 48^ the down-regulation of this isoform in the mPFC of CORT-treated hBDNF^Val/Val^ mice may serve to indirectly increase BDNF binding to fl.TrkB during activity-dependent processing. This result thus represents a novel effect of stress hormones upon the BDNF-TrkB signaling pathway that has not been previously described in this brain region as a result of BDNF genetic variation and stress exposure. In addition to a decrease in synaptophysin within the DHP of hBDNF^Val/Val^ mice (Figure 5a), expression of the postsynaptic marker PSD-95 within the mPFC also corresponded with the FST performance of the hBDNF^Val/Val^ group (Figure 5h). However, these subtle genotype-mediated changes in synaptic reorganization did not appear to influence NMDA receptor subunits (Figure 6), despite PSD-95 anchoring this receptor and previous studies finding that the Val66Met substitution alters synaptic physiology that requires this receptor in the hippocampus^50^ and infralimbic cortex.^51^ These experiments thus yield evidence of subtle synaptic reorganization as would be expected following a history of CORT or stress exposure, but implicate that these changes are mostly independent of the Val66Met variant.

Nonetheless, the current study has also yielded several surprising observations worthy of discussion. The first of these was a lack sex differences on the FST response, either as a product of prior CORT treatment or hBDNF^Val66Met^ genotype. This is incongruent with data that chronic CORT exposure may elicit sex-specific effects in rodents on the FST,^65^ although it is worth noting that not all studies have observed such differences.^66^ Nonetheless, the Val66Met polymorphism has been associated with sex-specific risk of depression,^67^ albeit inconsistently too (for review, see ref. 15). While we have observed sex-specific effects in our hBDNF^Val66Met^ polymorphic mice (for example, startle reactivity, see ref. 37, and other unpublished data not relevant to this manuscript), we do not observe differences for many other phenotypes (for example, hippocampus-dependent behavior, see ref. 31). This may suggest that the interaction between the BDNF^Val66Met^ polymorphism, stress and sex may be paradigm/phenotype specific, or gated by other factors. While we did not observe a sex-specific phenotype on the FST here, both at baseline and following CORT, this report is nonetheless a clue that the BDNF^Val66Met^ variant may influence risk of mood disorders via an effect on endogenous HPA axis homeostasis. It would be interesting for further investigations to examine whether hBDNF^Val66Met^ genotype alters endogenous HPA axis reactivity, both before and following a history of stress in male and female mice. Prior studies using this mouse line have reported no differences in CORT levels following restraint stress among females,^68^ however other reports have reported increases in circulating CORT, ACTH and hypothalamic CRH mRNA expression following restraint stress in BDNF^+/Met^ mice relative to wildtype controls.^25^ Data implicating genotype differences in response to early life stress,^69, 70^ as well as HPA axis reactivity have also been reported in humans.^69, 71, 72, 73^ While the aim of the current study was to evaluate and describe the long-term effects of chronic CORT exposure on the brain, it would be of interest to conduct an environmentally salient stress challenge or dexamethasone challenge following termination and wash-out of our CORT treatment to examine if lasting change in HPA-axis reactivity from prior stress are gated by hBDNF^Val66Met^ genotype, as this may provide additional insight into prior reports that hBDNF^Val66Met^ mice may be more vulnerable to stress in adulthood^31, 37^ well after the effects of CORT have washed-out.^33^

In summary, our data suggests that the BDNF^Val66Met^ polymorphism gates stress-related learned helplessness and that this is likely to occur via divergent pathways between the BDNF^Val/Val^ and BDNF^Met/Met^ genotype groups. While there may be overlap in these pathways, it appears there are separable effects of chronic CORT treatment between the two genotype groups. Our results implicate that TH expression within the mPFC and behavioral despair on the FST is disrupted in hBDNF^Met/Met^ mice at baseline, and that chronic CORT recapitulates these phenotypes in hBDNF^Val/Val^ wildtype mice. Further, we also identify that long-term and genotype-specific effects of chronic CORT treatment are more pronounced in hBDNF^Val/Val^ mice whereby, in addition to TH and behavioral despair, TrkB isoforms and select synaptic markers are impacted within the corticohippocampal axis. These data thus have important implications for the clinical literature, where an effect of the BDNF^Val66Met^ variant may be obscured should a history of stress not be stratified for and analyzed as a covariate. Furthermore, the molecular data reported here suggest that TH expression is perturbed in the mPFC of hBDNF^Met/Met^ mice, who have a robust despair phenotype irrespective of CORT treatment on the FST, and that in hBDNF^Val/Val^ mice chronic CORT is sufficient to recapitulate this TH expression profile and phenotype on the FST. This knowledge suggests that catecholamine innervation of the mPFC, likely by noradrenaline, may be a therapeutic target that can be exploited to treat depression-related symptoms in human BDNF^Val66Met^ carriers. In particular, these data suggest that it may be of value to more extensively trial the effectiveness of selective noradrenergic reuptake inhibitors on depression symptomology among BDNF^Val66Met^ carriers. As such, further research which seeks to delineate the effects of stress in BDNF^Val66Met^ carriers may eventually lead to advances in our knowledge of how common coding polymorphisms influence mood disorders, which could be utilized to predict risk and tailor intervention strategies.
